# The splicing factor SF3B4 drives proliferation and invasion in cervical cancer by regulating SPAG5

**DOI:** 10.1038/s41420-022-01120-3

**Published:** 2022-07-19

**Authors:** Yingwei Li, Yuchao Diao, Zixiang Wang, Shourong Wang, Jiali Peng, Beihua Kong

**Affiliations:** 1 Department of Obstetrics and Gynecology, Qilu Hospital of Shandong University, Jinan, Shandong 250012 China; 2 Medical Integration and Practice Center, Cheeloo College of Medicine, Shandong University, Jinan, Shandong 250012 China; 3 Department of Obstetrics and Gynecology, The Affiliated Hospital of Qingdao University, Qingdao, 266000 Shandong China

**Keywords:** Cervical cancer, Tumour biomarkers

## Abstract

Regulation of alternative splicing (AS) by the splicing factor 3b (SF3B) family plays an essential role in cancer. However, the biological function of SF3B family members in cervical cancer (CC) needs to be further elucidated. In this study, we found that splicing factor 3b subunit 4 (SF3B4) was highly expressed in CC by bioinformatics analysis using cervical squamous cell carcinoma and endocervical adenocarcinoma (CESC) data from The Cancer Genome Atlas (TCGA). Then, we demonstrated that high expression of SF3B4 promoted proliferation and invasion abilities of CC cells in vitro and in vivo and that reduced expression of SF3B4 performed the opposite effect. Further RNA-seq and AS analysis showed that sperm-associated antigen 5 (SPAG5) was a downstream target gene of SF3B4. Interestingly, SPAG5 expression was decreased after SF3B4 knockdown because of retained introns (RIs) and reduced maturation of SPAG5 pre-mRNA. Importantly, SPAG5 deficiency impaired the oncogenic effects of SF3B4 overexpression on CC cells. In conclusion, SF3B4 promotes CC progression by regulating the effective splicing of SPAG5. SF3B4 could be a promising target for CC.

## Introduction

Cervical cancer (CC) is a common gynecological malignancy in China [1]. Worldwide, approximately 311,365 patients died of CC in 2018, and its mortality in women ranks fourth among cancers [2]. In developing countries, the risk of death from CC is much higher than that in developed countries [3]. Moreover, there is an increasing trend of CC incidence in younger people [1, 4]. There have been great advances in the diagnostic and screening methods for CC in recent years, but the effectiveness of treatments for CC has not radically changed [5]. Following distant lymph node metastasis, the 5-year overall survival rate (OS) decreases significantly [6]. Hence, it is important to further study the molecular mechanism of CC development and search for new therapeutic targets for CC.

Alternative splicing (AS) is one of the major mechanisms of posttranscriptional regulation of genes, and it has been found in more than 95% of mammalian genes [7, 8]. RNA splicing requires a dynamic macromolecular ribonucleoprotein complex composed of multiple splicing factors (SFs) and five small nuclear ribonucleoproteins (snRNPs) [9], and it is the process by which introns are removed and exons of a pre-mRNA are connected to generate a mature mRNA [7, 10]. Dysregulation of AS induces several diseases, including cancer [8, 11–13]. Previous studies suggest that AS also plays an important role in CC [14–19]. For example, SRSF10 mediates IL1RAP alternative splicing and regulates CC progression [15]. ILF3 is regulated by SRSF3 and affects cell cycle progression in CC [20]. The splicing factor hnRNP mediates alternative splicing of HPV16 E6 to regulate the biological behavior of CC cells [14]. hnRNP A2/B1 can also affect CC cell proliferation and invasion through the PI3K/Akt pathway [21].

Members of the SF3B family participate in the regulation of pre-mRNA splicing and affect the initiation and development of many malignant tumors [22–27]. Importantly, small molecule inhibitors targeting SF3B have shown strong antitumor effects in several cancers, including endometrial cancer [22], CC [27], colorectal cancer [28] and other cancers [29, 30]. Therefore, we suspected that members of the SF3B family may also regulate AS events in CC and affect CC progression. Through analysis of the TCGA-CESC database, we found that only SF3B4 in the SF3B family was overexpressed in CC tissues. However, the effect of SF3B4 on CC is not clear.

In this study, we demonstrated that SF3B4 was overexpressed in CC patients. SF3B4 knockdown decreased the growth speed of CC cells in vitro and in vivo. Furthermore, SF3B4 regulated effective splicing of SPAG5 to promote CC progression. Therefore, SF3B4 might be a therapeutic target for CC treatment.

## Results

### SF3B4 is found to be overexpressed in cervical cancer

**Fig. 1:**
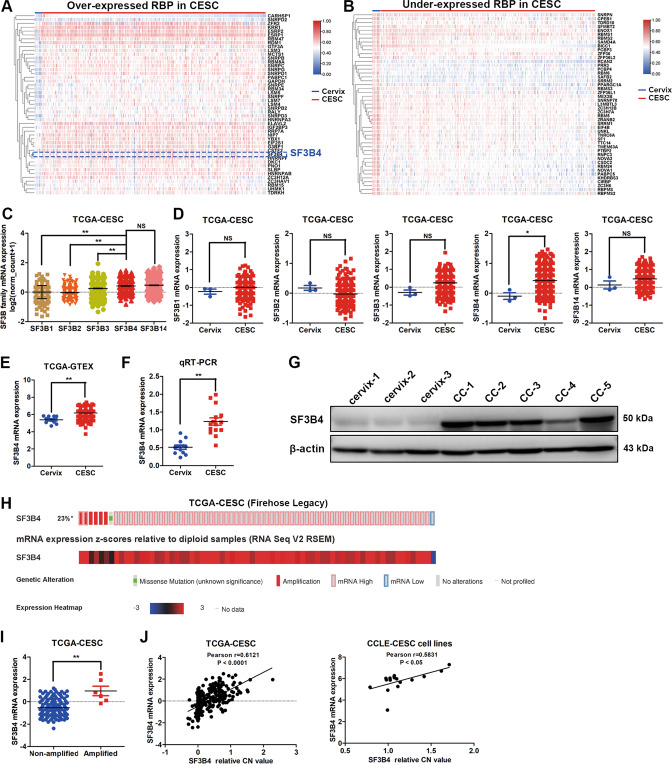
SF3B4 is overexpressed in cervical cancer. **A**, **B** Overlapping analysis of differentially expressed genes from the TCGA-CESC database and classical human RBPs from RBPDB (http://rbpdb.ccbr.utoronto.ca/). Representative overexpressed and downregulated RBPs are presented based on a heatmap. **C**, **D** Differential mRNA expression analysis of SF3B family members between cervical cancer (*n* = 305) and normal cervical tissues (*n* = 3) from TCGA-CESC database. **E** The differential mRNA expression of SF3B4 between cervical cancer (*n* = 306) and normal cervical (*n* = 10) tissues from the TCGA and GTEx databases. **F** qRT-PCR analysis of SF3B4 mRNA expression between normal cervix (*n* = 12) and cervical cancer fresh-frozen tissues (*n* = 15). **G** Western blot analysis of SF3B4 protein expression between normal cervix (*n* = 3) and cervical cancer (*n* = 5) fresh-frozen tissues. **H** The genomic and mRNA expression alteration of SF3B4 in TCGA-CESC (*n* = 310) from cBioPortal for Cancer Genomics (http://www.cbioportal.org/). **I** Differential mRNA expression analysis of SF3B4 in amplified samples (*n* = 136) compared with nonamplified samples (*n* = 6) in TCGA-CESC from cBioPortal for Cancer Genomics. **J** Correlation analysis between SF3B4 mRNA expression and relative copy number variation in TCGA-CESC (*n* = 292) and CCLE-CESC cell lines (*n* = 17). *P* value was obtained by Unpaired t-test. *P < 0.05, **P < 0.01.

To explore the critical splicing factors in CC, we performed overlap analysis of differentially expressed genes from the TCGA-CESC database and classical human RBPs from RBPDB (http://rbpdb.ccbr.utoronto.ca/). We found 47 overexpressed RBPs and more than 47 downregulated RBPs, and the results are presented in Fig. 1A, B. Current studies have shown that members of the SF3B family affect tumor initiation and development in several malignancies [22–25, 27, 31]. The differentially expressed RBP genes of SF3B family in CC are shown in Fig. 1A. SF3B4, an important subunit of SF3B, was found to be intimately with the poor prognosis and facilitated the proliferation and invasion abilities of ovarian cancer cells in our previous study. In this study, SF3B4 was found to be upregulated in CC. Then, we analyzed mRNA expression levels of SF3B family members in CC and found that the expression levels of SF3B4 and SF3B14 were the highest (Fig. 1C). Next, TCGA-CESC database was used to further analyze the mRNA expression of SF3B family members between CC and normal cervix, and we found that only SF3B4 expression was higher in CC tissues (Fig. 1D). The same result was observed in the TCGA and GTEx databases (Fig. 1E). SF3B4 was found to be overexpressed in fresh-frozen CC tissues comparing with normal cervix through qRT-PCR and western blot assays (Fig. 1F, G). Moreover, the expression of SF3B4 mRNA was increased in approximately 23% of CC cases (Fig. 1H). SF3B4 mRNA expression was higher in CC patients with copy number amplification than in nonamplified samples (Fig. 1I), and SF3B4 genomic amplification contributed to its high mRNA expression in cervical cancer tissues and cell lines (Fig. 1J). These results suggested that SF3B4 is overexpressed in CC, serving as a potential driver oncogene.

### SF3B4 promotes proliferation and motility of cervical cancer cells in vitro

**Fig. 2:**
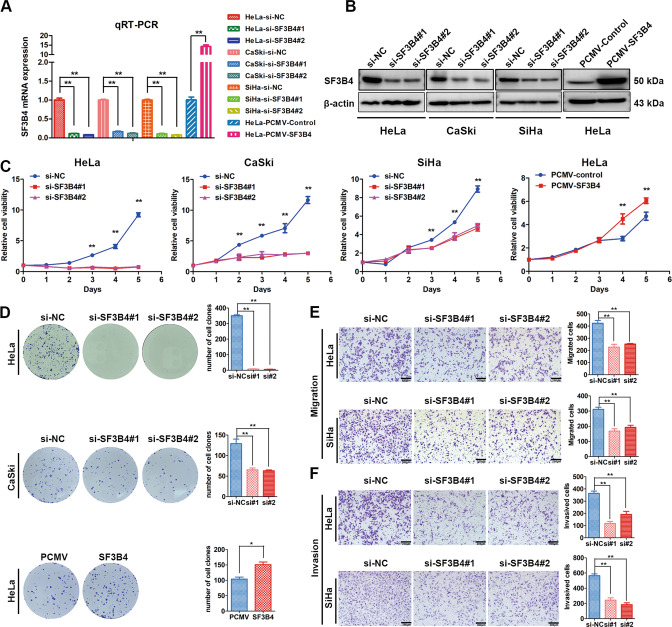
SF3B4 promotes the proliferation and motility of cervical cancer cells. **A**, **B** qRT-PCR and western blot analysis of SF3B4 mRNA and protein expression after transfection with SF3B4-siRNA and PCMV-SF3B4 vector in cervical cancer cells (*n* = 3 biologically independent samples). **C** Proliferation curve showing the impact of SF3B4 knockdown or overexpression on the growth ability of cervical cancer cells (*n* = 5 biologically independent samples). **D** The effect of SF3B4 knockdown or overexpression on the colony formation capacity of cervical cancer cells (*n* = 4 biologically independent samples). **E**, **F** Changes in migration and invasion abilities after SF3B4 downregulation in HeLa and SiHa cells (*n* = 3 biologically independent samples). *P* value was obtained by Unpaired t-test. *P < 0.05, **P < 0.01.

To clarify the effects of altered SF3B4 expression on CC cells, we established HeLa cell lines with stable SF3B4 overexpression. Then, we transiently transfected SF3B4-targeted siRNA into HeLa, CaSki and SiHa cells to knock down SF3B4 expression. qRT–PCR confirmed that SF3B4 mRNA was increased significantly after SF3B4 overexpression. In contrast, SF3B4 mRNA expression was downregulated after SF3B4 knockdown (Fig. 2A). The same results for protein expression were obtained by western blot analysis (Fig. 2B).

Next, we performed MTT and colony formation assays to evaluate the biological roles of SF3B4 on the growth ability of CC cells. Growth curve analysis showed that overexpression of SF3B4 notably promoted cell growth, whereas SF3B4 inhibition slowed proliferation of CC cells (Fig. 2C). The same results were observed in the colony formation assay (Fig. 2D). Furthermore, Transwell assays were applied to assess the effect of SF3B4 expression on the motility capacity of CC cells. As shown in Figs. 2E, F, SF3B4 downregulation notably weakened the CC cell migration and invasion abilities. Collectively, these results indicate that SF3B4 could promote the malignancy of CC cells in vitro.

### SF3B4 deficiency suppresses cervical cancer cell tumorigenesis in vivo

**Fig. 3:**
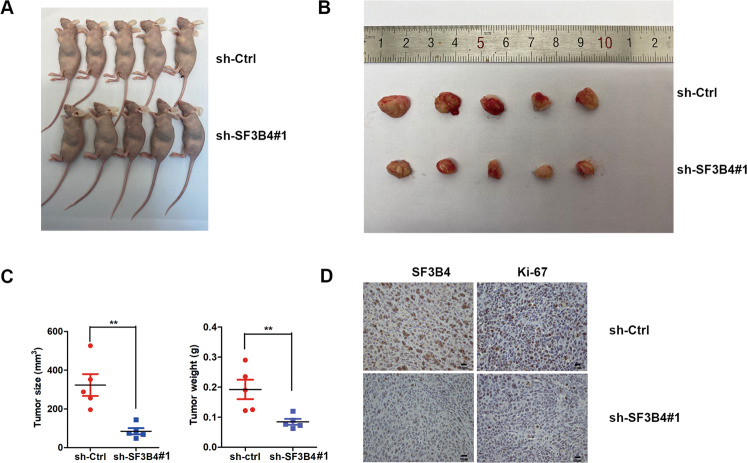
SF3B4 depletion suppresses xenograft tumorigenesis in vivo. **A** Images of xenograft tumors in mice (*n* = 5 per group). **B** Images of tumors isolated from nude mice implanted with sh-SF3B4#1 (*n* = 5) and sh-Ctrl (*n* = 5) CaSki cells. **C** Tumor weight and size in each group. **D** Images of immunohistochemical staining for SF3B4 and Ki-67 in xenograft tumor tissues. *P* value was obtained by Unpaired t-test. *P < 0.05, **P < 0.01.

As SF3B4 knockdown suppresses the proliferation and motility of CC cells through in vitro assays, we speculated that SF3B4 knockdown might also inhibit the tumorigenicity of CC cells in vivo. There was no obvious difference between siRNA#1 and siRNA#2 of SF3B4 based on the in vitro studies, and we constructed the SF3B4 shRNA based on the siRNA#1 sequences (http://www.addgene.org/protocols/plko/). Then, we generated CaSki cell lines with stable SF3B4 knockdown and control. CaSki cells expressing sh-SF3B4 and control were subcutaneously injected into nude mice. Three weeks later, all the mice were killed, and primary tumors were harvested (Fig. 3A, B). As shown in Fig. 3C, SF3B4 knockdown decreased the tumor size and tumor weight significantly, indicating that knockdown of SF3B4 expression efficiently reduced CC cell tumorigenesis in vivo. IHC staining of xenograft model tissue was applied to examine SF3B4 and Ki-67 proteins expression. The results indicated that the expression level of SF3B4 was significantly higher in the control group than that in the SF3B4 knockdown group. Ki-67 expression level was consistent with the expression of SF3B4 (Fig. 3D). These data suggested that SF3B4 could promote the proliferation of CC cells in vivo.

### SPAG5 is identified as a potential critical target of SF3B4 in cervical cancer

**Fig. 4:**
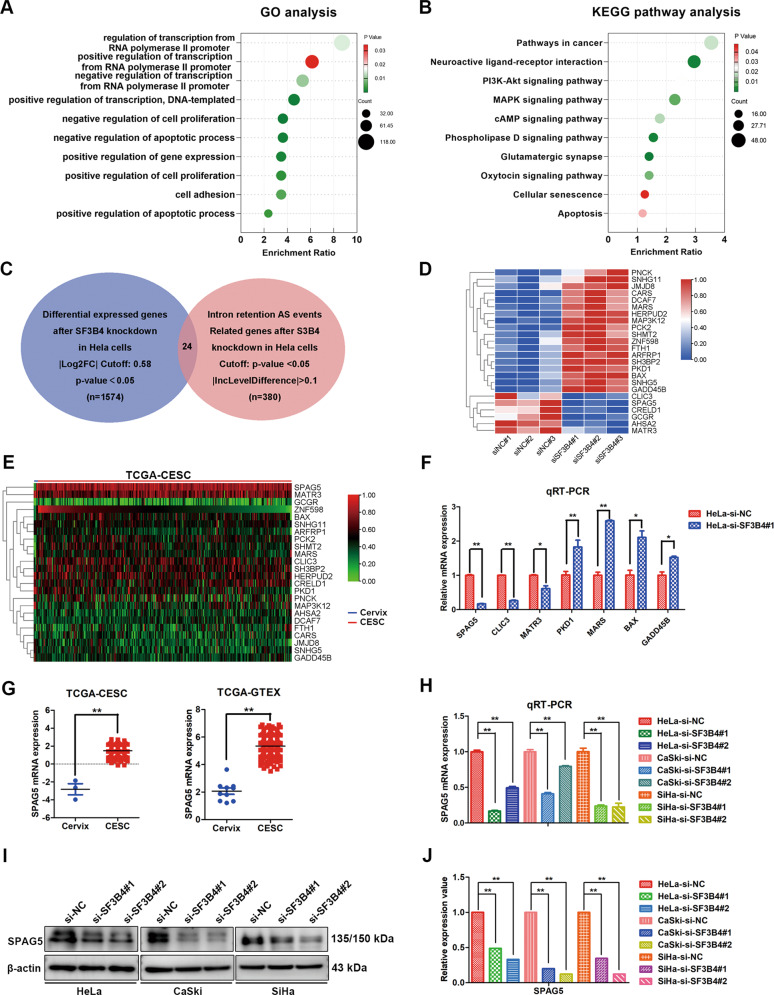
SPAG5 is identified as the critical downstream target of SF3B4 in cervical cancer. **A**, **B** Bubble diagram showing GO and KEGG pathway analysis of DEGs identified by RNA-seq after SF3B4 knockdown in HeLa cells. **C** Overlapping analysis of differentially expressed genes and intron retention-related genes after SF3B4 knockdown in HeLa cells. **D** Heatmap of 24 selected genes described in (**C**) from RNA-seq after SF3B4 knockdown in HeLa cells. **E** Differential mRNA expression analysis of 24 selected genes described in (**D**) between cervical cancer and normal cervical tissues in the TCGA-CESC cohort from UCSC xena (https://xena.ucsc.edu/). **F** qRT-PCR analysis of the mRNA expression of selected DEGs described in (**E**) (*n* = 3 biologically independent samples). **G** Differential mRNA expression analysis of SPAG5 in the TCGA-CESC (CESC, *n* = 305; normal cervix, *n* = 3) and TCGA-GTEx (CESC, *n* = 306; normal cervix, *n* = 10) databases. **H**, **I** qRT-PCR and western blot analysis showed SPAG5 mRNA and protein expression changes after SF3B4 knockdown in HeLa, CaSki and SiHa cells (*n* = 3 biologically independent samples). **J** Relative protein expression change of SPAG5 upon SF3B4 knockdown in cervical cancer cells. *P* value was obtained by Unpaired t-test. *P < 0.05, **P < 0.01.

To investigate the mechanism by which SF3B4 affects CC proliferation and motility, we performed RNA-seq on SF3B4-knockdown HeLa cells. Gene Ontology analysis (https://david.ncifcrf.gov/) showed that the differentially expressed genes (DEGs) were enriched in the following biological processes: regulation of transcription, regulation of cell proliferation, regulation of apoptotic process and cell adhesion (Fig. 4A). Based on KEGG pathway analysis, DEGs were enriched in pathways involved in cancer, PI3K-Akt, MAPK signaling and cAMP signaling pathways (Fig. 4B). rMATS was applied to analyze alternative splicing (AS) events after SF3B4 knockdown based on the results of RNA-seq. Among them, splicing events related to intron retention were changed after SF3B4 knockdown compared with the control group. Then, we took the intersection of 1574 DEGs and 380 intron retention AS event-related genes after SF3B4 knockdown in HeLa cells, and 24 genes were identified (Fig. 4C, D). Next, we used the TCGA-CESC database to analyze the differential mRNA expression of these 24 genes between CC and normal cervical tissues (Fig. 4E) and found that most were differentially expressed. Subsequently, qRT-PCR was used to determine the DEGs in HeLa cell lines after SF3B4 knockdown. The results revealed that SPAG5, CLIC3 and MATR3 were significantly downregulated and that PKD1, MARS, BAX and GADD45B were upregulated (Fig. 4F), which was consistent with the RNA-seq results. Combined with the analysis results shown in Fig. 4E, F, these results indicate that SPAG5 mRNA expression had the most change. Therefore, SPAG5 was selected for further research.

SPAG5 mRNA expression was found to be higher in CC than in normal cervical tissues using the data from the TCGA-CESC and TCGA-GTEx databases (Fig. 4G). To further verify the regulatory effect of SF3B4 on SPAG5, the changes of SPAG5 mRNA and protein expressions were assessed through qRT-PCR and western blot assays after silencing SF3B4 expression in CC cells. The results showed that the mRNA and protein levels of SPAG5 decreased notably after reduced SF3B4 expression (Fig. 4H–J). In summary, SPAG5 is an important downstream target gene of SF3B4 in CC.

### SF3B4 promotes the alternative splicing of SPAG5 mRNA

**Fig. 5:**
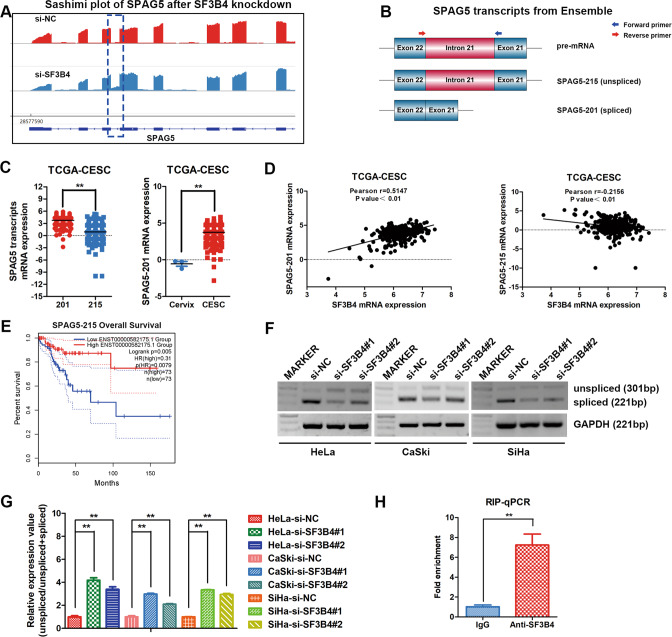
SF3B4 promotes efficient splicing of SPAG5. **A** Sashimi plot visualizing RNA-seq reads of SPAG5 after SF3B4 knockdown in HeLa cells. **B** Schematic diagram showing two SPAG5 mRNA transcripts (SPAG5-201 and SPAG5-215) from the Ensemble genome browser and the position of primers used for RT–PCR. **C** Differential mRNA expression analysis of SPAG5 transcripts (SPAG5-201 and SPAG5-215) in the TCGA-CESC (*n* = 306) database. Differential mRNA expression analysis of the SPAG5-201 transcript between cervical cancer (*n* = 306) and normal cervical tissues (*n* = 3) from TCGA-CESC database. **D** Correlation analysis between SPAG5 transcript mRNA expression and SF3B4 mRNA expression (*n* = 306). **E** Kaplan–Meier analysis showed the effect of SPAG5-215 expression on the overall survival of cervical cancer patients from TCGA-CESC using the online GEPIA website (High expression group, *n* = 73; Low expression group, *n* = 73) (http://gepia.cancer-pku.cn/). **F** RT–PCR analysis of SPAG5 spliced and unspliced transcripts after SF3B4 knockdown in cervical cancer cells (*n* = 3 biologically independent samples). **G** Relative mRNA expression of SPAG5 transcripts (unspliced/spliced) upon SF3B4 knockdown in cervical cancer cells. **H** The interaction between the SF3B4 protein and SPAG5 mRNA was validated by RIP-PCR in HeLa cells (*n* = 3 biologically independent samples). *P* value was obtained by Unpaired t-test. *P < 0.05, **P < 0.01.

To prove whether SF3B4 regulates SPAG5 expression by alternative splicing, the RNA-seq reads of SPAG5 were visualized on a Sashimi plot in response to SF3B4 knockdown in HeLa cells, and intron 21 retention of SPAG5 was found to be increased (Fig. 5A). The transcripts of SPAG5 mRNA were analyzed based on the Ensemble genome browser, and an intron (intron 21) was found to be retained in the unspliced transcript SPAG5–215 compared to the spliced transcript SPAG5–201 (Fig. 5B). Retained intron 21 of SPAG5 could produce stop codons, resulting in premature termination of codon in the coding region of SPAG5. SPAG5–201 mRNA expression level was obviously higher than that of SPAG5–215 in CC tissues, and SPAG5–201 mRNA expression was also higher in CC tissues (Fig. 5C). Then, we performed correlation analysis between SF3B4 and SPAG5 transcript mRNA expression using the data from TCGA-CESC database. Interestingly, SPAG5–201 transcript expression was positively correlated with SF3B4 mRNA expression. In contrast, SPAG5–215 transcript expression was negatively associated with SF3B4 expression (Fig. 5D). In addition, we found that the SPAG5–215 transcript was associated with longer survival time in CC patients (Fig. 5E).

RT–PCR was performed to verify the relative changes in SPAG5 transcript (SPAG5–201 and SPAG5–215) levels. We designed specific primers within exons 21 and 22 to span intron 21, and the results showed that unspliced transcript SPAG5–215 was increased and spliced transcript SPAG5–201 was decreased distinctly after SF3B4 downregulation (Fig. 5F, G). More importantly, RIP-PCR revealed that the expression of SPAG5 mRNA in the SF3B4 precipitates was much higher than that in the IgG control precipitates (Fig. 5H), indicating that the SPAG5 mRNA could bind to the SF3B4 protein. Combining these results with the above results, we concluded that SF3B4 regulates the alternative splicing of SPAG5 via intron retention.

### Knockdown of SPAG5 decreases the growth and motility of cervical cancer cells

**Fig. 6:**
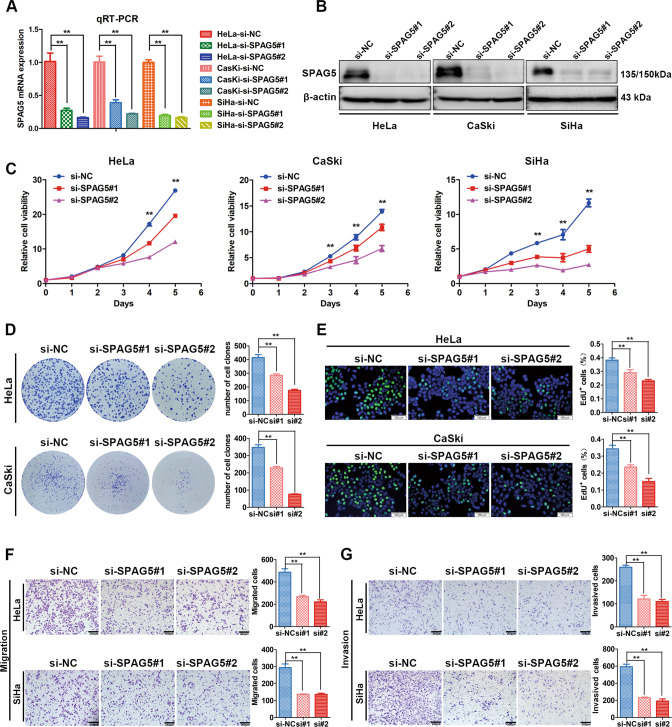
Knockdown of SPAG5 suppresses the proliferation, migration and invasion of cervical cancer cells in vitro. **A**, **B** qRT-PCR and western blot analysis showed the interference efficiency of SPAG5-targeted siRNA in cervical cancer cells (*n* = 3 biologically independent samples). **C** Effect of SPAG5 knockdown on the growth ability of cervical cancer cells (*n* = 5 biologically independent samples). **D** Effect of SPAG5 knockdown on the colony formation capacity of HeLa and CaSki cells (*n* = 4 biologically independent samples). **E** EdU incorporation assay of changes in the proliferation ability after SPAG5 knockdown in cervical cancer cells (*n* = 4 biologically independent samples). **F**, **G** Transwell assays were performed to assess the effect of SPAG5 on the migration and invasion abilities of HeLa and SiHa cells (*n* = 3 biologically independent samples). *P* value was obtained by Unpaired t-test. *P < 0.05, **P < 0.01.

To investigate biological effect of SPAG5 on CC, SPAG5-targeted siRNA was used to knockdown SPAG5 expression in CC cells. SPAG5 mRNA and protein expression levels were reduced significantly in CC cells after transient transfection with SPAG5 siRNAs through qRT-PCR and western blot assays (Fig. 6A, B).

Then, we evaluated the impact of SPAG5 knockdown on the biological functions of CC cells. The MTT assay demonstrated that SPAG5 knockdown inhibited the proliferation capacity of CC cells (Fig. 6C). Similarly, SPAG5 deficiency decreased colony formation ability of CC cells (Fig. 6D). The same results were observed in the EdU incorporation assay (Fig. 6E). Subsequently, the effect of altered SPAG5 expression on the motility of CC cells was evaluated by Transwell assays. SPAG5 knockdown markedly impaired metastasis ability of CC cells (Fig. 6F, G). These results imply that SPAG5 can drive the growth and metastasis of CC cells.

### SPAG5 deficiency reduced the biological effects of SF3B4 overexpression

**Fig. 7:**
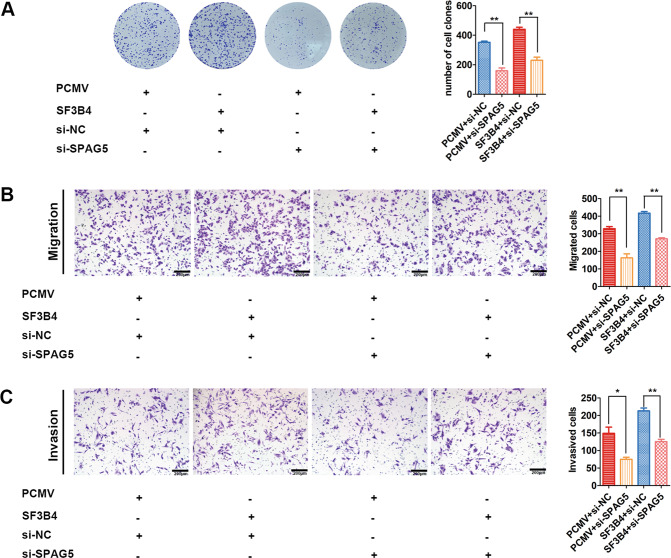
Depletion of SPAG5 impairs the promotive effects of SF3B4. **A** Colony formation assays showed the effect of SPAG5 knockdown on changes in the proliferation ability of SF3B4-overexpressing HeLa cells (*n* = 4 biologically independent samples). **B**, **C** Transwell assays were performed to assess the effect of SPAG5 deficiency on the migration and invasion abilities of HeLa cells stably overexpressing SF3B4 (*n* = 3 biologically independent samples). *P* value was obtained by Unpaired t-test. *P < 0.05, **P < 0.01.

**Fig. 8:**
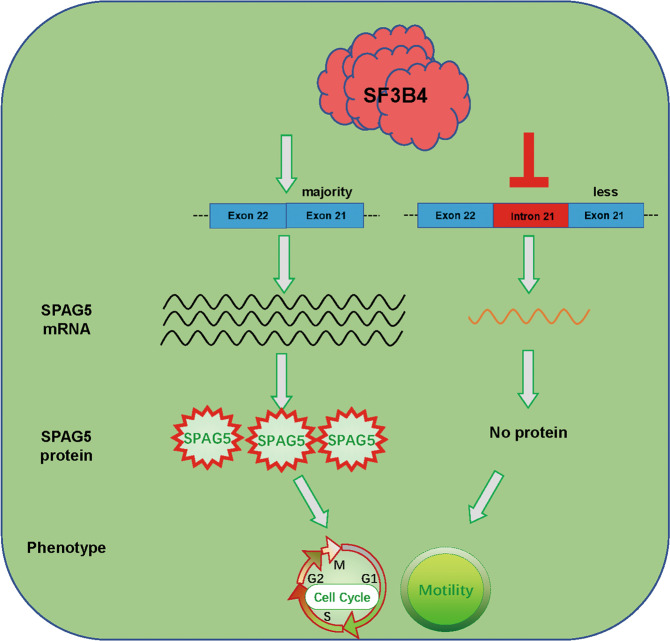
Model showing the effect of SF3B4/SPAG5 signaling on the malignancy of cervical cancer cells. The splicing factor SF3B4 drives malignant behavior in cervical cancer by regulating the normal splicing of SPAG5. When SF3B4 is deficient, SPAG5 splicing becomes abnormal and its expression is decreased.

A rescue experiment was performed to further prove whether SF3B4 increased the malignancy of CC cells by regulating SPAG5 expression. We established stable SF3B4 overexpression (PCMV-SF3B4) and control cell lines (PCMV-control) in HeLa cells. Then, we transiently transfected siNC or siSPAG5 into PCMV-SF3B4 and control HeLa cells. The results of colony formation assays indicated that knockdown of SPAG5 expression suppressed the abilities of SF3B4 overexpression promoting colony formation (Fig. 7A). Similarly, SPAG5 knockdown also impaired the capacity of SF3B4 to facilitate metastasis (Fig. 7B, C). These results implied that SF3B4 promotes the malignant behavior of CC cells in a SPAG5-dependent manner (Fig. 8).

## Discussion

Alternative splicing allows the production of different transcripts that encode multiple proteins from a single gene. For this reason, a limited number of genes can produce a vast diversity of proteins in human [32, 33]. Many studies have demonstrated that the level of AS increases in cancer, and cancer cells have cancer type-specific splicing alterations that can affect cancer progression [8, 19]. Increasing evidence has revealed that splicing abnormalities was associated with various cancers, such as breast cancer [34], ovarian cancer [35, 36], lung cancer [37], prostate cancer [23], endometrial cancer [22], glioma [38], renal cancer [24], colorectal cancer [39], and hepatocellular cancer [40]. Previous studies also suggest that AS affects the progression and prognosis of CC [14–20].

SF3B family members bind to the U2 snRNP and regulates the AS of pre-mRNA [41], impacting the progression and prognosis of many cancers [22–25, 27]. Therefore, we speculate that abnormalities in the SF3B family may affect the molecular mechanism of CC initiation and progression. Then, we found by analysis of the TCGA-CESC database that only SF3B4 in the SF3B family was significantly upregulated in CC. Next, the effect of SF3B4 on CC cells was determined through in vitro and in vivo assays. The results found that SF3B4 knockdown inhibited the growth speed and xenograft tumorigenic ability of CC cells. These results indicate that SF3B4 is an oncogene in CC.

Posttranscriptional regulation of RNAs is a key step in gene expression control. AS is one of the crucial posttranscriptional regulation mechanisms [16]. AS analysis of our RNA-seq database showed that RI-associated splicing events were significantly changed after SF3B4 knockdown in HeLa cells. RIs always lead to the formation of premature termination codons (PTCs), and these transcripts are degraded through nonsense-mediated mRNA decay (NMD) or translated into dysfunctional proteins [42–44]. Several biological processes could be regulated by RIs, including cell cycle [45], centriole duplication [46], and cellular senescence [47]. In this study, intron 21 of SPAG5 mRNA was found to be retained after SF3B4 knockdown, inducing the reduced maturation of SPAG5 pre-mRNA. Combined with the DEG analysis of the TCGA-CESC database and AS analysis of RNA-seq data after SF3B4 knockdown in HeLa cells, we found that SPAG5 mRNA expression was overexpressed in CC tissues and SF3B4 knockdown decreased the expression of SPAG5. Hence, we considered SPAG5 as a critical downstream target gene for further study.

SPAG5 is a mitotic spindle-binding protein that involves in regulating the process of mitosis [48–50]. SPAG5 is involved in regulating the progression of multiple cancers. SPAG5 regulates breast cancer progression by regulating cell cycle and correlates with poor prognosis [51, 52]. SPAG5 alters sensitivity to Taxol via the mTOR signaling pathway in CC [53]. SPAG5 promotes hepatocellular cancer proliferation and acts as an oncogene [54, 55]. Similarly, our data demonstrated that deficiency of SPAG5 reduces the proliferation and motility of CC cells.

In conclusion, our study confirmed that SF3B4 was upregulated in CC tissues. SF3B4 promoted the malignant behavior of CC cells by enhancing their proliferation and invasion capacities. Moreover, SF3B4 knockdown decreased the splicing efficiency of oncogenic SPAG5, resulting in decreased expression of SPAG5. SPAG5 knockdown impaired the biological function of SF3B4 overexpressing in CC cells. Taken together, these findings indicate that SF3B4 functions as an oncogene in CC by regulating SPAG5. Thus, the SF3B4/SPAG5 axis constitutes a potential therapeutic target for CC patients in the future.

## Materials and methods

### Patients and tissue samples

CC and normal cervix tissues were from patients who underwent gynecological surgical excision at Qilu Hospital of Shandong University. CC specimens were obtained from patients who had not previously undergone treatment. Normal cervix tissues were obtained from the patients who underwent full hysterectomy and bilateral salpingectomy and judged by the pathologist. Ethics recognition was approved by the Ethics Committee of Shandong University School of Medicine (SDULCLL2019-1-09).

### Bioinformatics analysis

The differentially expressed genes data between CC and normal cervix tissues of TCGA was from GEPIA (|Log2FC|≥1, *q* < 0.01) (http://gepia.cancer-pku.cn/). The DEGs data between CC and normal tissues of TCGA-GTEX was from UCSC Xena (http://xena.ucsc.edu/). The genomic alteration of SF3B4 was analyzed through online cBioPortal [56] (http://www.cbioportal.org/). The correlation analysis of SF3B4 between genomic amplification and mRNA expression in CC tissues and cell lines was from cBioPortal and Cancer Cell Line Encyclopedia (CCLE)(https://sites.broadinstitute.org/ccle/datasets).

Gene Ontology and KEGG pathway analysis of DEGs were performed by David 6.8 (https://david.ncifcrf.gov/). Venny 2.1.0 online tool was used to perform overlapping analysis (https://bioinfogp.cnb.csic.es/tools/venny/). Sashimi plot tool was used to visualize the RNA-seq reads mapped to SPAG5 after SF3B4 knockdown. SPAG5 transcripts were from Ensemble website (https://asia.ensembl.org/index.html).

### Cell lines and cell culture

HeLa, CaSki, SiHa and HEK293T cells were from Chinese Academy of Sciences (Shanghai, China). HeLa, SiHa and HEK293T cells were cultured in the DMEM containing 10% fetal bovine serum (FBS). CaSki cells were cultured in the medium of RPMI-1640 containing 10% FBS. All the cell lines were identified by short tandem repeat (STR) analysis.

### RNA interference, plasmid transfection and lentiviral infection

We purchased SF3B4- and SPAG5-targeted small interfering RNAs (siRNA#1 and 2) from GenePharma (Shanghai, China). The SF3B4 and SPAG5 siRNAs were transfected into CC cells using Lipofectamine 2000 (Invitrogen, 11668–019). RNA and protein samples were extracted 48 h after transfection. The siRNAs used in this study are shown in Table S1.

SF3B4 open reading frames (ORFs) were purchased from Vigenebio (Jinan, China, CH804949) and cloned into pCMV vector (OriGene, PS100069). SF3B4 short hairpin RNA (shRNA) designed based on siRNA#1 was synthesized by Sangon Biotech (Shanghai, China) and cloned into pLKO.1 vector (Addgene, 10879). The cell lines were generated by infecting with lentiviral particles for 24 h and selecting them with puromycin (2 µg/ml) for 7 days to obtain the stable transfection cell lines. The shRNA sequences are shown in Table S1.

### RNA isolation, qRT–PCR and RT–PCR

We extracted total RNA using TRIzol (Invitrogen, 15596018) and obtained cDNA using PrimeScript RT master mix kits (Takara, RR037A). SYBR Green qPCR master mix (Takara, RR420A) was used to perform qRT–PCR assay. The 2^−ΔΔCT^ method was applied to analyze relative expressions of targets. We used Phusion High-Fidelity PCR Master Mix (BioLabs, M0531s) to perform RT–PCR. ImageJ software was used to analyze the relative expression of genes. GAPDH was served as endogenous control. The primers used in this study are shown in Table S2.

### Protein extraction and western blotting (WB)

Protein lysates of cells and tissues were obtained using RIPA (Beyotime Bio, P0013). The protein concentration was calculated by a BCA Protein Assay Kit (Merck Millipore, 71287). Proteins were separated by SDS–PAGE and transferred to the 0.22 µm PVDF membrane. The PVDF membrane was blocked with 5% skim milk. The membrane was incubated with the primary antibody at 4 °C overnight followed by. Then, the membrane was detected with an ECL system after incubation with a secondary antibody. β-Actin was used as the endogenous control. Primary antibodies used in this study included anti-SF3B4 (Proteintech, 10482–1-AP) and anti-SPAG5 (Proteintech, 14726–1-AP) antibodies.

### Cell proliferation assay (MTT)

CC cells were seeded into 96-well plates and cultured for 5–6 days. MTT solution (Sigma–Aldrich) was added to each well and incubated for 4 h. The culture medium was removed, and added 100 µl of DMSO (Sigma–Aldrich) each well. Finally, a microplate spectrophotometer (Thermo Scientific) was used to measure the absorbance at 450 nm.

### EdU incorporation assay

An EdU Kit (Beyotime, C0071s) was used to apply the EdU incorporation assay (EdU). 1.5 × 10^4^ CC cells/well were seeded into 96-well plates and cultured with EdU reagent (1:1000 dilution) for 2 h the next day. Then, 4% paraformaldehyde was used to fix the cells, and fluorescent dye and Hoechst were used to stain cells. Photoshop software was used to count EdU-positive cells.

### Colony formation assay

1000 CC cells/well were seeded in 6-well plates and cultured for 10–14 days. Then, the cells were fixed by methanol and stained by 0.6% crystal violet. Cell clones (more than 50 cells) were counted using Photoshop software.

### Transwell assays

1 × 10^5^ CC cells were seeded into the upper compartment of the chamber and culture medium containing 20% FBS was added into the lower compartment. Methanol was used to fix cells, and 0.6% crystal violet was used to stain cells. Photoshop software was used to count migrated and invaded cells.

### Tumor formation assay in nude mice

Female BALB/c nude mice (5 weeks old) were from GemPharmatech (Nanjing, China) and fed under SPF conditions. CaSki cells (5 × 10^6^) transfected with pLKO.1-control and pLKO.1-shSF3B4 were subcutaneously injected into the axilla of each mouse. Three weeks after injection, all the mice were killed, and the tumors were harvested. Then, the tumors were photographed, and the weight and volume (length × width^2^ / 2) of the tumors were measured. Immunohistochemistry staining was used to evaluate SF3B4 and Ki-67 expression levels in xenograft tumors. All the animal experiments were approved by the Shandong University Animal Care Committee.

### Immunohistochemical (IHC) staining

Four-micrometer-thick sections were used for IHC staining. The sections were deparaffinized and rehydrated with xylene and a graded series of ethanols. Then, the slides were incubated using the primary antibodies (SF3B4, Proteintech, 10482–1-AP and Ki-67, Cell Signaling Technology, 9449T) at 4 °C overnight after antigen retrieval. Finally, a DAB detection system (ZSGB-BIO, Beijing, China) was used to detect staining.

### RNA-seq and bioinformatic analysis

We used TRIzol reagent to extract RNA from HeLa cells transiently transfected using SF3B4 siRNA and control. Each sample of different groups was repeated 3 times independently. RNA-seq (Annoroad Genomics Co., Ltd, China) was performed to analyze the differences between the two groups. |log2FoldChange | ≥0.58 and *p* < 0.05 were established as the significance criteria.

### RNA immunoprecipitation assay (RIP)

RIP assays were applied using a RIP Kit (Guangzhou Geneseed Biotech, P0101) to analyze the interaction between SF3B4 protein and SPAG5 mRNA. qRT–PCR was used to detect SPAG5 mRNA expression level in the immunoprecipitated complex.

### Statistical analysis

Statistical significance between two different groups was calculated using Student’s t test. Results represent the mean ± SD of three independent experiments. Experimental data *P* < 0.05 (*P < 0.05, **P < 0.01) was considered statistically significant.

## Supplementary information

Supplementary tables

Original Data File

## Data Availability

The datasets used and/or analyzed during the current study are available from the corresponding author on reasonable request.
