# Supplementary material for: The splicing factor SF3B4 drives proliferation and invasion in cervical cancer by regulating SPAG5
Source: Cell Death Discov. 2022 Jul 19;8:326. doi: 10.1038/s41420-022-01120-3 (PMC9296558; doi:10.1038/s41420-022-01120-3)
Supplement: Supplementary file 1 — Supplementary tables [file 41420_2022_1120_MOESM1_ESM.docx]

**Supplementary tables**

**Supplementary table 1. si-RNA and shRNA sequences used in this study.**

**Supplementary table 2. Primer sequences used in this study.**

**Supplementary table 1. si-RNA and shRNA sequences used in this study.**

| Method | Name | Sequence (5’-3’) |
| --- | --- | --- |
| si-RNA | si-SF3B4#1 | GGAUGAGAAGGUUAGUGAATT |
| si-RNA | si-SF3B4#2 | GCACCAAGGCUAUGGCUUUTT |
| si-RNA | si-SPAG5#1 | GGACUUAGUACCUUCUGAATT |
| si-RNA | si-SPAG5#2 | GGUGUAAAUACCUCCGUCATT |
| si-RNA | negative control | UUCUCCGAACGUGUCACGUTT |
| sh-RNA | sh-SF3B4-F1 | CCGGGGATGAGAAGGTTAGTGAACCCTCGAGGGTTCACTAACCTTCTCATCCTTTTTG |
| sh-RNA | sh-SF3B4-R1 | AATTCAAAAAGGATGAGAAGGTTAGTGAACCCTCGAGGGTTCACTAACCTTCTCATCC |
|  |  |  |

**Supplementary table 2. Primer sequences used in this study.**

| Method | Name | Sequence (5’-3’) |
| --- | --- | --- |
| qPCR | GAPDH-F | GGTCTCCTCTGACTTCAACA |
| qPCR | GAPDH-R | GTGAGGGTCTCTCTCTTCCT |
| qPCR | SF3B4-F | AGTCAACACCCACATGCCAA |
| qPCR | SF3B4-R | CACCCGTATTGGCTTCCCAT |
| qPCR | SPAG5-F | GCATGGAGCTATGGAGGAAAG |
| qPCR | SPAG5-R | TCAGAGCCAAGAATCCTACCAA |
| qPCR | CLIC3-F | CCTCAAGGGCGTACCTTTCAC |
| qPCR | CLIC3-R | GTCGCTGTCATAGAGCAGGA |
| qPCR | MATR3-F | CAGCAGTCTACAAATCCAGCACC |
| qPCR | MATR3-R | CTGCATGTGTCTAGGTCCTTGC |
| qPCR | PKD1-F | AGCAGCACGGTCACCATTCCAC |
| qPCR | PKD1-R | CACTCCAAGGACACAATGGGCA |
| qPCR | MARS-F | GGCAAGCTCATCAATGCTGTCG |
| qPCR | MARS-R | AACCACTCCTCCAGTCGCTTCT |
| qPCR | BAX-F | TCAGGATGCGTCCACCAAGAAG |
| qPCR | BAX-R | TGTGTCCACGGCGGCAATCATC |
| qPCR  qPCR | GADD45B -F  GADD45B -R | GCCAGGATCGCCTCACAGTGG  GGATTTGCAGGGCGATGTCATC |
| RT-PCR | SPAG5-F | GAGAGGAGGTGACCCACCTTA |
| RT-PCR | SPAG5-R | GCTCTGGAACTTGATCATGAGTTT |
