# Supplementary figures and images for: The splicing factor SF3B4 drives proliferation and invasion in cervical cancer by regulating SPAG5
Source: Cell Death Discov. 2022 Jul 19;8:326. doi: 10.1038/s41420-022-01120-3 (PMC9296558; doi:10.1038/s41420-022-01120-3)

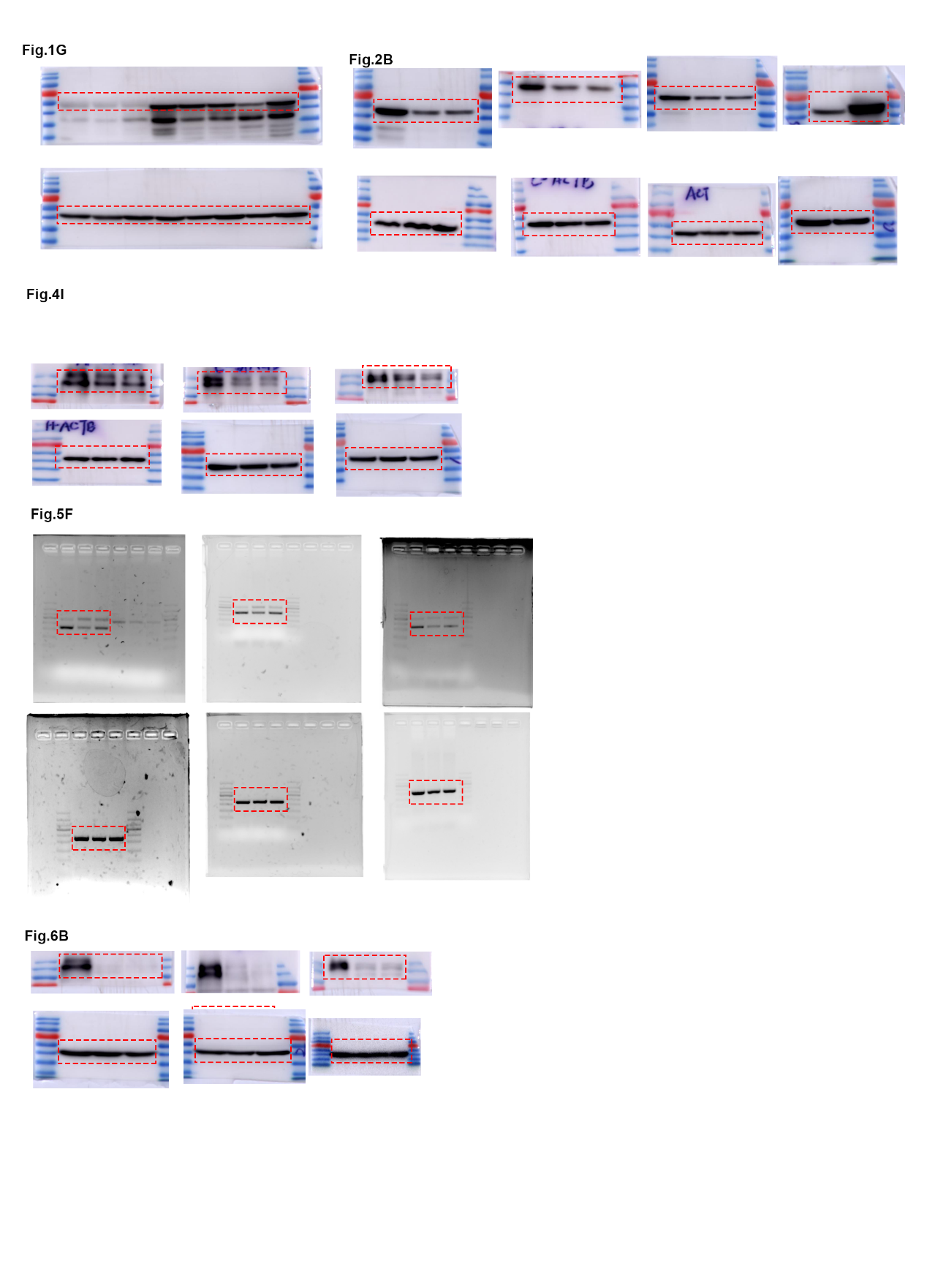

Supplement: Supplementary file 2 — Original Data File [file 41420_2022_1120_MOESM2_ESM.docx]
